# Sucrase–Isomaltase Deficiency in Children with Functional Gastrointestinal Disorders

**DOI:** 10.3390/jcm15103639

**Published:** 2026-05-09

**Authors:** Firdevs Kavas Demirci, Tuğba Gürsoy Koca, Abdulkerim Elmas, Mustafa Akçam

**Affiliations:** 1 Department of Pediatrics, Pamukkale University Faculty of Medicine, 20160 Denizli, Türkiye; kvs.firdevs@gmail.com; 2 Division of Pediatric Gastroenterology, Hepatology and Nutrition, Department of Pediatrics, Pamukkale University Faculty of Medicine, 20160 Denizli, Türkiye; tgkoca@gmail.com; 3 Division of Pediatric Gastroenterology, Hepatology and Nutrition, Department of Pediatrics, Suleyman Demirel University Faculty of Medicine, 32260 Isparta, Türkiye; makcam32@gmail.com

**Keywords:** sucrase-isomaltase deficiency, functional gastrointestinal disorders, irritable bowel syndrome, child, disaccharidase deficiency, quality of life

## Abstract

**Background:** Congenital sucrase–isomaltase deficiency (CSID) may mimic functional gastrointestinal disorders (FGIDs) and is likely underrecognized in pediatric practice. This study aimed to determine the frequency of sucrase–isomaltase (SI) gene variants among children with FGIDs and to evaluate genotype–phenotype associations and treatment-related quality-of-life outcomes. **Methods:** In this prospective cross-sectional study, children aged 0–18 years diagnosed with FGIDs according to Rome IV criteria were enrolled between May 2022 and January 2023. All patients underwent next-generation sequencing for SI gene variants. Clinical characteristics, FGID subtypes, and anthropometric data were recorded. Variant-positive patients received dietary sucrose restriction, and selected patients were treated with sacrosidase enzyme replacement. Symptom severity was assessed using the Numeric Rating Scale, and quality of life was evaluated with the Pediatric Quality of Life Inventory (PedsQL 4.0). **Results:** Among 290 children with FGIDs, SI gene variants were identified in 17 patients (5.9%). Variants were more frequently detected in children with irritable bowel syndrome–like symptoms. Clinical presentation was heterogeneous, and no consistent genotype–phenotype correlation was observed. Dietary intervention was associated with symptom improvement in compliant patients, while sacrosidase therapy led to significant improvements in both child- and parent-reported PedsQL scores. **Conclusions:** Sucrase–isomaltase deficiency is not uncommon among children with FGIDs and should be considered, particularly in those with IBS-like symptoms or diet-related complaints. Integrating genetic evaluation with targeted dietary and enzyme-based therapy may improve symptom control and quality of life in selected pediatric patients.

## What Is Already Known

Congenital sucrase–isomaltase deficiency (CSID) may present with symptoms that overlap with functional gastrointestinal disorders (FGIDs), particularly irritable bowel syndrome–like complaints.

Variants in the sucrase–isomaltase gene have been associated with IBS in adult populations, but pediatric data remain limited.

Dietary sucrose restriction and sacrosidase enzyme replacement can improve gastrointestinal symptoms in selected patients with CSID.

## What This Study Adds

This study provides the largest prospective pediatric cohort from Türkiye evaluating the frequency of sucrase–isomaltase gene variants among children diagnosed with FGIDs according to Rome IV criteria.

Sucrase–isomaltase variants were identified in a substantial proportion of children with IBS-like symptoms, supporting the consideration of CSID in the differential diagnosis of pediatric FGIDs.

Targeted dietary intervention and sacrosidase therapy were associated with improvements in symptom severity and quality of life, highlighting the clinical value of a personalized diagnostic and therapeutic approach.

## 1. Introduction

Disaccharidases are brush-border enzymes responsible for hydrolyzing disaccharides into monosaccharides. Their deficiency leads to intraluminal accumulation of undigested carbohydrates, increased osmotic load, gas production, and intestinal distension, which may cause early satiety and abdominal pain, particularly in individuals with visceral hypersensitivity [1]. Functional gastrointestinal disorders (FGIDs), now referred to as disorders of gut–brain interaction (DGBI) are characterized by chronic, recurrent gastrointestinal symptoms without identifiable structural or biochemical abnormalities and are diagnosed based on symptom criteria defined by Rome IV [2,3]. Congenital sucrase–isomaltase deficiency (CSID), first described in 1960, is caused by mutations in the sucrase–isomaltase gene [4]. Although historically considered rare, its reported prevalence has increased from approximately 0.05–0.2% in earlier European studies to as high as 2–9% in more recent reports [5].

CSID should be considered in the differential diagnosis of patients with functional gastrointestinal symptoms, particularly those with persistent or refractory complaints. CSID may present with IBS-like symptoms, including diarrhea, abdominal bloating, and abdominal pain. Adult studies have reported a higher prevalence of sucrase–isomaltase gene variants among patients with IBS, with heterozygous carrier status associated with increased IBS risk [6,7].

Several diagnostic methods are available for disaccharidase deficiencies, including breath tests, genetic analyses, and measurement of enzyme activity in endoscopic biopsies. Although biopsy-based assessment is considered the gold standard, its invasive nature limits routine use. Consequently, genetic testing for sucrase–isomaltase variants has emerged as a less invasive and promising diagnostic approach, particularly in patients with IBS-like symptoms [6,7].

Disaccharidase deficiencies in children are increasingly recognized and may coexist within the same patient. The heterogeneous clinical presentation of CSID can delay diagnosis and is associated with impaired growth and reduced quality of life. Dietary modification and enzyme replacement therapy have been shown to effectively control symptoms [8,9].

This study aimed to determine the frequency of CSID among children diagnosed with functional gastrointestinal disorders according to Rome IV criteria at the Pediatric Gastroenterology outpatient clinic of Pamukkale University Faculty of Medicine and to evaluate genotype–phenotype associations and treatment-related quality-of-life outcomes in patients with identified genetic variants.

## 2. Materials and Methods

This single-center, prospective cross-sectional study included children aged 0–18 years presenting with FGIDs symptoms to the Pediatric Gastroenterology outpatient clinic of Pamukkale University Faculty of Medicine between May 2022 and January 2023. FGID diagnoses were established according to Rome IV criteria. Patients with known organic gastrointestinal diseases, structural abnormalities, or previously diagnosed metabolic or genetic disorders were excluded. Sucrase–isomaltase gene analysis was performed by next-generation sequencing of coding exons and exon–intron boundaries using genomic DNA extracted from peripheral blood. Library preparation was carried out with the FAST SI NGS Sequencing Kit (Multigen, Istanbul, Türkiye), and sequencing was performed on the Illumina MiniSeq platform (Illumina Inc., San Diego, CA, USA). Variant classification was performed according to the American College of Medical Genetics and Genomics (ACMG) guidelines. Variants were categorized as pathogenic, likely pathogenic, variants of uncertain significance (VUS), likely benign, or benign based on available evidence from population databases (GnomAD), disease databases (HGMD, ClinVar), and in silico prediction tools (VarSome, Franklin). For patients with identified variants, demographic data, clinical features, dietary habits, laboratory findings, and anthropometric measurements were recorded. Anthropometric status was assessed using WHO criteria, with weight-for-height SDS calculated for children ≤5 years and BMI SDS for those >5 years.

Symptom severity was evaluated using the Numeric Rating Scale (NRS). Variant-positive patients were advised to follow a sucrose-restricted diet, and those with persistent severe symptoms received sacrosidase enzyme replacement therapy. Treatment response was assessed using NRS and the Pediatric Quality of Life Inventory (PedsQL 4.0) before treatment and at the second week of follow-up. For children under 4 years of age, the parent-proxy version of the PedsQL 4.0 was used, as self-reporting is not applicable in this age group.

Statistical analyses were performed using SPSS v25.0. Appropriate parametric or nonparametric tests were applied, and *p* < 0.05 was considered statistically significant. The study was approved by the Non-Interventional Clinical Research Ethics Committee of Pamukkale University (17 May 2022; approval no. 08).

## 3. Results

**Figure 1:**
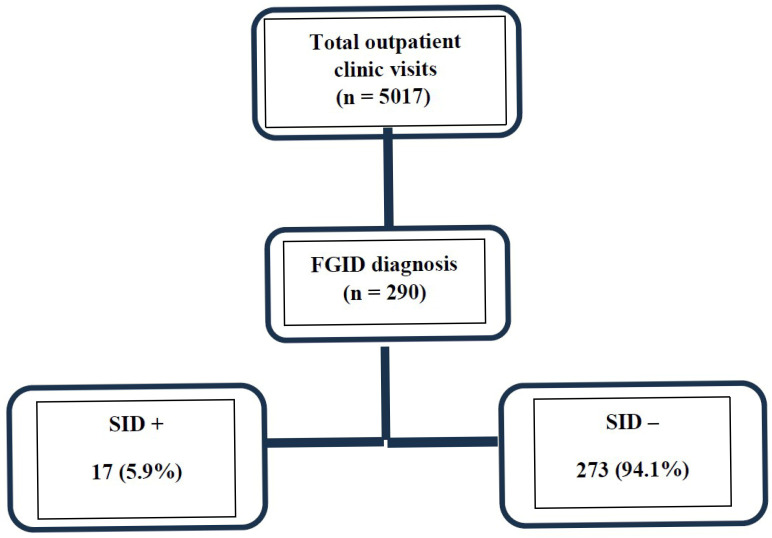
Flowchart of patient selection. Abbreviations: FGID, functional gastrointestinal disorder; SID, congenital sucrase–isomaltase deficiency.

During the study period, 5017 patients were evaluated in the pediatric gastroenterology outpatient clinic. Among them, 290 children diagnosed with FGIDs according to Rome IV criteria underwent sucrase–isomaltase gene analysis. SI gene variants, all classified as variants of uncertain significance, were identified in 17 patients (5.9%). Therefore, this subgroup does not represent the entire FGID population (Figure 1).

The mean age of the cohort was 9.3 ± 5.2 years, and 53.8% were female. Among variant-positive patients, the mean age was 10.9 ± 5.1 years, and 70.6% were female. No significant differences were observed between patients with and without identified variants regarding age, sex, anthropometric measurements, or malnutrition prevalence (Table 1).

Sucrase–isomaltase variants were more frequently detected in patients with IBS-like symptoms, and the combination of abdominal pain, diarrhea, and constipation was significantly more common among variant-positive patients (*p* = 0.022) (Table 2). According to Rome IV criteria, diarrhea-predominant IBS was the most frequent IBS subtype. Abdominal migraine was significantly more prevalent in variant-positive patients, whereas functional abdominal pain was more common in variant-negative patients.

According to the Rome IV criteria, 110 (37.9%) patients were diagnosed with functional abdominal pain, 59 (20.3%) with IBS, 42 (14.5%) with functional dyspepsia, 35 (12.1%) with functional diarrhea, 20 (6.9%) with functional vomiting, 8 (2.8%) with functional constipation, 5 (1.7%) with abdominal migraine, 5 (1.7%) with functional nausea, 2 (0.7%) with aerophagia, and 2 (0.7%) with infantile colic.

Clinical presentation among variant-positive patients was heterogeneous, even among individuals carrying the same mutation, and no consistent genotype–phenotype correlation was identified (Table 3). All of the variants identified were categorised as VUS. None of the variants in the patients included in the analysis were classified as benign or likely benign. All variant-positive patients were advised to follow a sucrose-restricted diet; compliant patients showed reduced symptom severity. One asymptomatic patient (V8) was included due to family screening, as this individual was a sibling of a variant-positive case (V7). Although this patient did not meet criteria for FGIDs, genetic testing was performed to evaluate potential familial segregation.

Five patients with identified SI gene variants received sacrosidase enzyme replacement therapy. Health-related quality of life was assessed using the PedsQL 4.0 before treatment and at 2 weeks of follow-up. Significant improvements were observed in physical functioning and total PedsQL scores in child self-reports (*p* = 0.043 for both), whereas changes in emotional, social, and school functioning scores were not statistically significant. Similarly, parent-reported outcomes demonstrated significant improvements in physical functioning (*p* = 0.043), emotional functioning (*p* = 0.042), and total scores (*p* = 0.043), while no significant changes were observed in social and school functioning domains. The degree of improvement in total scores varied among patients, with individual increases ranging from 2.6% to 63.7% (Table 4).

## 4. Discussion

This study represents the largest prospective investigation evaluating the frequency of congenital sucrase–isomaltase deficiency among pediatric patients diagnosed with functional gastrointestinal disorders in Türkiye. The limited number of studies on this topic in our country may be attributable to insufficient recognition of CSID and its omission from the differential diagnosis. In our cohort of patients diagnosed with FGIDs according to the Rome IV criteria, the frequency of SI gene variants was 5.9%, suggesting that CSID is not a rare condition in childhood.

The true prevalence of congenital sucrase–isomaltase deficiency in the Turkish population remains unknown. To date, only a single case report and a case series comprising four patients with CSID have been published from Türkiye [10,11]. In previously reported studies, the frequency of sucrase–isomaltase gene mutations among pediatric patients diagnosed with nonspecific chronic diarrhea or chronic diarrhea has ranged from 6.8% to 11.7% [12,13]. In contrast to these reports, our study evaluated all FGID subtypes rather than focusing solely on diarrhea, allowing the observed prevalence to reflect a broader clinical spectrum. From this perspective, our findings indicate that CSID is not limited to chronic diarrhea and may also be present across different FGID subgroups.

The overlap between disaccharidase deficiencies and FGID symptoms constitutes a major challenge in diagnosis. In pediatric patients with disaccharidase deficiencies, abdominal pain, bloating, gas, diarrhea, and dyspeptic symptoms may predominate; however, the symptom profile often does not allow reliable clinical prediction of a specific enzyme deficiency [9,14]. Indeed, studies evaluating children with chronic abdominal pain using endoscopic biopsy to measure disaccharidase activity have reported that a substantial proportion have at least one disaccharidase deficiency, while clinical features alone were of limited value in predicting the specific deficiency [9,14]. These findings are consistent with the heterogeneous clinical presentation observed in our study.

In recent years, increasing attention has been directed toward the association between IBS and variants in the sucrase–isomaltase (SI) gene. Henström et al. demonstrated that functional variants in the SI gene are associated with an increased risk of IBS, whereas Garcia-Etxebarria et al. reported that heterozygous carriage of rare pathogenic or loss-of-function SI variants may also be linked to IBS [6,7]. In our study, the identification of SI gene variants in 10.1% of patients meeting the Rome IV criteria for IBS suggests that SID/CSID should be considered in the differential diagnosis of pediatric patients with IBS-like symptoms, particularly those with suspected dietary triggers or symptoms refractory to standard therapeutic approaches.

With regard to growth impairment, classical descriptions of congenital sucrase–isomaltase deficiency emphasize that prolonged malabsorption and chronic symptoms may contribute to impaired growth [1,14]. Reported rates of growth retardation in pediatric cohorts with chronic diarrhea from Türkiye also point to the clinical burden associated with delayed diagnosis [12,13]. In our study, the presence of malnutrition in both FGID patients with and without SI gene variants suggests that growth impairment may not be attributable solely to SI deficiency; decreased appetite, selective eating behaviors, dietary restrictions, and accompanying psychosocial factors related to FGIDs may also play important roles. Nevertheless, the persistence of symptoms and prominent diet-related complaints in patients with identified SI variants indicate that closer growth monitoring may be warranted in this subgroup.

The fact that a substantial proportion of the genetic findings in our study were classified as VUS reflects a challenge that is also frequently emphasized in the current literature: while some variants in the SI gene may be functionally relevant, their clinical expression can vary considerably among individuals [15,16]. Therefore, diagnostic decision-making should not rely solely on genetic results; instead, the symptom profile, dietary history, response to dietary interventions, and, when feasible, functional confirmation through breath testing or measurement of disaccharidase activity in biopsy specimens should be evaluated in conjunction. A key limitation of our genetic findings is that all the variants identified have been classified as VUS. This situation limits the ability to draw definitive conclusions regarding the pathogenic role of these variants and their direct contribution to enzyme deficiency. As there are no functional validation methods available, such as disaccharidase activity measurement or breath tests, these variants must be interpreted with caution. Therefore, the presence of SI gene variants in our cohort should be interpreted as indicating a potential genetic susceptibility rather than a confirmed diagnosis of CSID.

With respect to diagnostic approaches, measurement of disaccharidase activity in endoscopic biopsy specimens is traditionally considered the reference method; however, its invasive nature and limited accessibility preclude its use in all patients. Recent reviews suggest that, in the presence of clinical suspicion, less invasive strategies—such as genetic analyses, breath tests, and/or therapeutic trials—may offer practical value, particularly in selected patient subgroups [14,15,17]. Although the genetic screening approach used in our study contributes to this practicality, the absence of confirmation by direct measurement of disaccharidase activity limits genotype–phenotype interpretation. Given the limited number of variant-positive patients, genotype–phenotype analysis in this study should be considered exploratory. The observed heterogeneity in clinical presentation, even among patients carrying the same variant, further highlights the complexity of establishing reliable genotype–phenotype correlations in the absence of functional validation.

From a therapeutic perspective, symptom improvement with a sucrose-restricted diet is consistent with previous reports; however, dietary adherence is often challenging, and dietary modification alone may not be sufficient in all patients [14,15,16]. The efficacy of sacrosidase enzyme replacement therapy has been demonstrated in a randomized, double-blind study in the pediatric population, showing that it can facilitate dietary liberalization and contribute to symptom control in appropriately selected patients [18]. In our study, the observed improvement in PedsQL scores among patients receiving sacrosidase supports the notion that enzyme replacement therapy may have beneficial effects not only on symptom reduction but also on overall quality of life. This finding underscores the importance of a targeted therapeutic approach in a subset of patients labeled as having FGIDs but who present with diet-related or treatment-refractory symptoms. However, these findings should be interpreted with caution, as the follow-up period was relatively short and no control group was included. Given the fluctuating nature of symptoms in functional gastrointestinal disorders, part of the observed improvement may reflect natural variation rather than a direct treatment effect.

The limitations of this study include its single-center design and the limited number of patients with identified SI variants, which reduced the statistical power of certain subgroup analyses. The most significant limitation of this study is that SID has not been functionally confirmed. The diagnosis of CSID is traditionally based on the detection of reduced disaccharidase activity in intestinal biopsy samples or, alternatively, on a breath test. In our study, the interpretation is based on genetic analysis, which does not provide direct evidence of enzyme dysfunction. Therefore, it cannot be considered sufficient to establish a definitive diagnosis of CSID. Consequently, the identified variants should be interpreted as indicators of possible genetic predisposition rather than as confirmation of clinically significant enzyme deficiency. As not all genetic variants lead to enzymatic dysfunction or symptomatic disease, this distinction is important. These limitations may lead to an overestimation of the clinical significance of SI gene variants in children with FGID. Therefore, our findings should be interpreted with caution and should not be used alone to establish a diagnosis without supporting functional or clinical evidence. Nevertheless, the use of standardized Rome IV criteria, the evaluation of a broad spectrum of FGIDs, and the assessment of treatment response in conjunction with quality-of-life measures represent notable strengths of the study.

## 5. Conclusions

In conclusion, in children presenting with FGIDs, the possibility of sucrase–isomaltase gene variants/SID may be considered, particularly in those with IBS-like symptoms, diet-triggered complaints, or limited response to standard therapeutic approaches. The development of selective testing strategies guided by clinical suspicion and the conduct of multicenter studies to better delineate genotype–phenotype relationships may help reduce unnecessary investigations and facilitate targeted treatment, thereby improving quality of life.

## Figures and Tables

**Table 1:** Demographic and anthropometric characteristics of the patients.

		SID+ *n* = 17	SID− *n* = 273	*p*
**Sex**	Female (*n*, %)	12 (70.6)	144 (52.7)	0.152 *
	Male (*n*, %)	5 (29.4)	129 (47.3)	
**Age**	Mean ± SD	10.882 ± 5.11	9.205 ± 5.145	0.192 ^φ^
	Median (IQR)	11 (7–15)	9 (5–14)	
	Min–max	2–18	1–18	
**Body weight** **SDS**	Mean ± SD	0.077 ± 2.064	−0.338 ± 1.551	0.296 ^ω^
	Median (IQR)	−0.67 (−1.6–1.21)	−0.39 (−1.3–0.6)	
**Height SDS**	Mean ± SD	−0.091 ± 1.403	−0.217 ± 1.336	0.884 ^ω^
	Median (IQR)	−0.14 (−1.26–0.84)	−0.24 (−0.91–0.63)	
**Body mass index SDS**	Mean ± SD	−0.049 ± 1.774	−0.322 ± 1.548	0.485 ^ω^
	Median (IQR)	−0.61 (−1.465–1.37)	−0.19 (−1.29–0.67)	

Abbreviations: SID, sucrase–isomaltase deficiency; SDS, standard deviation score; SD, standard deviation; IQR, interquartile range; *n*, number of patients. * Chi-square test; ^φ^ Independent samples *t*-test; ^ω^ Mann–Whitney U test.

**Table 2:** Distribution of genetic variants according to presenting symptoms.

		SID+ *n* = 17	SID− *n* = 273	*p*
**Presenting symptom**	Nausea, (*n*, %)	0 (%0)	5 (%1.8)	-
	Belching, (*n*, %)	0 (%0)	2 (%0.7)	-
	Irritability, (*n*, %)	0 (%0)	2 (%0.7)	-
	**Diarrhea**, (*n*, %)	**2 (%11.8)**	33 (%12.1)	-
	Constipation, (*n*, %)	0 (%0)	8 (%2.9)	-
	**Abdominal pain**, (*n*, %)	**7 (%41.2)**	**112 (%41)**	-
	**Abdominal pain + diarrhea**, (*n*, %)	**2 (%11.8)**	31 (%11.4)	-
	**Abdominal pain + diarrhea + constipation,** (*n*, %)	**4 (%23.5)**	16 (%5.9)	**0.022**
	Abdominal pain + constipation, (*n*, %)	0 (%0)	6 (%2.2)	-
	Vomiting, (*n*, %)	**1 (%5.9)**	20 (%7.3)	-
	Bloating, (*n*, %)	0 (%0)	38 (%13.9)	0.14

Categorical variables were compared using the chi-square test or Fisher’s exact test, as appropriate. *p* < 0.05 was considered statistically significant, *p* values are shown only for comparisons with sufficient cell counts. Abbreviations: SID, sucrase–isomaltase deficiency. Bold *p* values indicate statistical significance.

**Table 3:** Clinical characteristics, mutation types, and treatment status of patients with identified variants.

	Age (Years)	Growth Retardation	Presenting Symptom	FGID Diagnosis	Mutation Type	Endoscopy	Treatment ^¥^
V1	2	-	Diarrhea	Functional diarrhea	Y975H	-	-
V2	2	-	Diarrhea	Functional diarrhea	Y975H	-	-
V3	9	-	Abdominal pain + diarrhea + constipation	IBS-M	T964T	-	**+**
V4	15	-	Abdominal pain + diarrhea	IBS-D	T964T	-	**+**
V5	10	-	Abdominal pain	Functional dyspepsia	c.4841 + 1G > A	-	-
V6	18	-	Abdominal pain + diarrhea + constipation	IBS-M	V371M	-	**+**
V7	15	-	Abdominal pain + diarrhea + constipation	IBS-M	Y1650X	**+**	-
V8	6	+	None	None	Y1650X	-	-
V9	17	+	Abdominal pain + diarrhea + constipation	IBS-M	T964T	**+**	**+**
V10	18	+	Abdominal pain	Abdominal migraine	D1028A	**+**	**+**
V11	14	+	Abdominal pain	Functional dyspepsia	D1028A	-	-
V12	14	-	Abdominal pain	Abdominal migraine	Y975H	-	-
V13	7	+	Abdominal pain	Functional dyspepsia	c.4841 + 1G > A	-	-
V14	7	+	Abdominal pain	Functional dyspepsia	V1651I	-	-
V15	8	+	Vomiting	Functional vomiting	V1651I	-	-
V16	12	+	Abdominal pain	Functional abdominal pain	Y1525Y	-	-
V17	11	-	Abdominal pain + diarrhea	IBS-D	V1188I	-	-

V8: asymptomatic sibling identified through family screening. Abbreviations: FGID, functional gastrointestinal disorder; IBS, irritable bowel syndrome; IBS-D, diarrhea-predominant IBS; IBS-M, mixed-type IBS. ^¥^ Sacrosidase enzyme replacement therapy. “+” denotes presence or yes, whereas “-“ denotes absence or no.

**Table 4:** Comparison of pre-treatment and post-treatment PedsQL outcomes in patients receiving therapy and their parents.

	Pre-Treatment Mean ± SD	Post-Treatment Mean ± SD	*p*	Change
Physical Functioning-patients Physical Functioning-parents	71.24 ± 17.02 72.48 ± 19.57	82.49 ± 14.43 81.86 ± 18.17	0.043 * (z = −2.023) 0.043 * (z = −2.023)	18.82 ± 25.86 14.3 ± 10.06
Emotional Functioning-patients Emotional Functioning-parents	45 ± 32.79 51 ± 32.09	56 ± 31.7 63 ± 29.28	0.109 (z = −1.604) 0.042 * (z = −2.032)	34.32 ± 48.04 36.1 ± 37.75
Social Functioning-patients Social Functioning-parents	78 ± 25.88 88 ± 21.68	84 ± 22.47 88 ± 21.68	0.18 (z = −1.342) 1 (z = 0)	11.04 ± 15.13 0 ± 0
School Functioning-patients School Functioning-parents	69 ± 22.47 73 ± 17.18	87 ± 14.4 86 ± 9.62	0.068 (z = −1.826) 0.066 (z = −1.841)	33.58 ± 35.48 20.58 ± 16.76
Total Score-patients Total Score-parents	263.22 ± 61.66 284.46 ± 53.2	308.46 ± 49.36 318.84 ± 50.68	0.043 * (z = −2.023) 0.043 * (z = −2.023)	20.1 ± 24.75 12.54 ± 4.02

* *p* < 0.05 indicates statistical significance; Mean: arithmetic mean; SD: standard deviation; z: Wilcoxon signed-rank test.

## Data Availability

The data presented in this study are available on request from the corresponding author.
